# Associations Between State-Level Severe Maternal Morbidity and Other Perinatal Indicators

**DOI:** 10.1001/jamanetworkopen.2022.24621

**Published:** 2022-07-28

**Authors:** Ashley H. Hirai, Pamela L. Owens, Lawrence D. Reid, Catherine J. Vladutiu, Elliott K. Main

**Affiliations:** 1 Office of Epidemiology and Research, Maternal and Child Health Bureau, Health Resources and Services Administration, US Department of Health and Human Services, Rockville, Maryland; 2 Center for Financing, Access and Cost Trends, Agency for Healthcare Research and Quality, US Department of Health and Human Services, Rockville, Maryland; 3 California Maternal Quality Care Collaborative, Stanford University, Palo Alto

## Abstract

This cross-sectional study examines state-level associations between severe maternal morbidity and other perinatal indicators, including prepregnancy hypertension, prepregnancy diabetes, prepregnancy obesity, low-risk cesarean delivery, preterm birth, and maternal and infant mortality.

## Introduction

Severe maternal morbidity (SMM) during delivery hospitalizations is a leading indicator of maternal health in the US that is assessed from hospital discharge data using an algorithm of *International Classification of Diseases, Ninth Revision,* diagnosis and procedure codes.^1^ Previous individual-level validation studies^2,3^ show reasonable positive predictive value compared with medical record review of near-miss criteria in a single state or hospital, particularly when blood transfusion is excluded. To assess the validity and comparability of state-level SMM rates, we examined state-level associations between SMM and other perinatal indicators that may contribute to (eg, prepregnancy hypertension, diabetes, and obesity and low-risk cesarean delivery), coincide with (eg, preterm birth), or result from (eg, maternal and infant mortality) SMM.^4,5,6^

## Methods

State-level SMM rates per 10 000 delivery hospitalizations were calculated on the basis of patient state of residence using revised code sets for 20 indicators excluding blood transfusion^1^ from the 2017 to 2019 Healthcare Cost and Utilization Project’s State Inpatient Databases (HCUP-SID). The HCUP-SID include all discharge records from short-term, nonfederal, community hospitals for 47 states and the District of Columbia.

As a secondary analysis of anonymized data, the Agency for Healthcare Research and Quality human protections administrator determined this project did not constitute research involving human participants; thus, informed consent and institutional review board approval were not required. The Strengthening the Reporting of Observational Studies in Epidemiology (STROBE) reporting guidelines for cross-sectional studies were followed.

Contemporaneous perinatal indicators, also by state of residence, were obtained using the HCUP-SID codes for prepregnancy hypertension (H35.03x, I10-I16.x, I97.3, and O10.x-O11.x) and prepregnancy diabetes (E08.x, E10.x-E13.x, O24.0x-O24.3x, O24.8x-O24.9x, and O99.81x) and from the National Vital Statistics Systems’ (NVSS) birth and death certificate data for prepregnancy obesity (body mass index [weight in kilograms divided by height in meters squared] ≥30), low-risk cesarean delivery (nulliparous, term, singleton, and vertex), preterm birth (<37 weeks), infant mortality per 1000 live births (<1 year), and maternal mortality per 100 000 live births (during or within 42 days of the end of pregnancy). Given the small numbers, maternal mortality rates (MMRs) were examined with 5 years of data (2016-2020) using the 2018 coding method to reduce pregnancy checkbox errors. All states had NVSS data, 44 had reportable MMRs (≥10 deaths), and 42 also had HCUP-SID data.

Spearman rank correlation coefficients between state-level SMM rates and indicators were calculated using SAS statistical software version 9.4 (SAS Institute). To examine geographical patterning, state-level SMM and MMRs were mapped using ArcGIS mapping software version 10.6.1 (Esri). Significance was determined using Fisher *Z* transformation 2-sided tests and *P* < .05. Data analysis was performed from April 2021 to March 2022.

## Results

From 2017 to 2019, 10 542 942 maternal deliveries from HCUP-SID and 11 394 752 live births from NVSS were aggregated for state-level analysis. SMM rates were significantly correlated with 2 of 7 other perinatal indicators: prepregnancy hypertension (*r* = 0.39; 95% CI, 0.12-0.61; *P* = .005) and low-risk cesarean delivery rates (*r* = 0.36; 95% CI, 0.08-0.58; *P* = .01). All other perinatal indicators were significantly associated with at least 4 of 7 other indicators, and most correlations were higher in magnitude (*r* ≥ 0.50) (Table). SMM rates exhibited little geographical patterning, with high rates observed on both coasts. MMRs were highest in the southeast (Figure).

**Table.** Spearman Rank Correlations Between State-Level Perinatal Indicators, 2017-2019

Indicator	*r* (95% CI)							
	Severe maternal morbidity	Prepregnancy hypertension	Prepregnancy diabetes	Prepregnancy obesity	Low-risk cesarean delivery	Preterm birth	Infant mortality	Maternal mortality^a^
Severe maternal morbidity	1	0.39 (0.12 to 0.61)	0.06 (–0.23 to 0.34)	–0.28 (–0.53 to 0.00)	0.36 (0.08 to 0.58)	0.04 (–0.24 to 0.32)	–0.07 (–0.35 to 0.22)	–0.25 (–0.52 to 0.05)
Prepregnancy hypertension		1	0.57 (0.33 to 0.73)	0.48 (0.23 to 0.68)	0.51 (0.26 to 0.69)	0.68 (0.48 to 0.81)	0.65 (0.45 to 0.79)	0.42 (0.13 to 0.64)
Prepregnancy diabetes			1	0.57 (0.34 to 0.73)	0.01 (–0.27 to 0.29)	0.52 (0.28 to 0.70)	0.50 (0.25 to 0.69)	0.27 (–0.04 to 0.53)
Prepregnancy obesity				1	0.15 (–0.13 to 0.41)	0.56 (0.33 to 0.72)	0.66 (0.48 to 0.79)	0.54 (0.48 to 0.79)
Low-risk cesarean delivery					1	0.49 (0.25 to 0.68)	0.31 (0.04 to 0.54)	0.32 (0.02 to 0.56)
Preterm birth						1	0.82 (0.71 to 0.90)	0.59 (0.35 to 0.75)
Infant mortality							1	0.56 (0.32 to 0.74)
Maternal mortality								1

^a^ Maternal mortality rates used 5-year data (2016-2020) because of small numbers.

**Figure.**
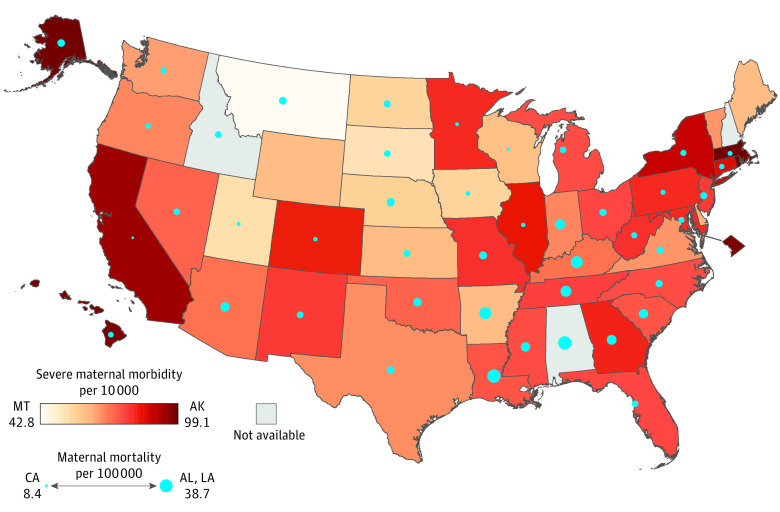
Severe Maternal Morbidity and Maternal Mortality Rates by State Severe maternal morbidity per 10 000 delivery hospitalizations was determined from the Healthcare Cost and Utilization Project–State Inpatient Databases, 2017 to 2019. State rates of severe maternal morbidity are displayed in a sequential red color scheme, with darker red indicating higher rates. Maternal mortality per 100 000 live births was determined from the National Vital Statistics System, 2016 to 2020; a checkbox to indicate pregnancy on the death certificate was not in use or did not follow national standards for California (all years) and West Virginia (2016 to mid-2017). State rates of maternal mortality are displayed in proportional blue circles with larger circles indicating higher rates.

## Discussion

In this cross-sectional study, state SMM rates (excluding blood transfusion) were not consistently associated with other perinatal indicators that should be related, particularly maternal mortality. SMM was originally constructed using conditions associated with maternal mortality,^5^ but the current findings may indicate a need for further measure refinement. Several SMM indicators have shown poor positive predictive value relative to expert clinical review of the medical record.^2^ Coding consistency may also vary across states but could not be directly assessed in this ecological study. Future research could compare the accuracy of medical record documentation and billing coding across states, perhaps among a subset of easily identifiable severe cases with in-hospital death or intensive care unit stays of at least 24 hours. Until more is known, SMM comparisons across states should be made with caution.
